# Adapting to the Coronavirus Pandemic: Building and Incorporating a Diagnostic Pipeline in a Shared Resource Laboratory

**DOI:** 10.1002/cyto.a.24248

**Published:** 2020-11-22

**Authors:** Emma Russell, Ana Agua‐Doce, Lotte Carr, Asha Malla, Kerol Bartolovic, Dina Levi, Carl Henderson, Debipriya Das, Hefin Rhys, Philip Hobson, Sukhveer Purewal, Andrew Riddell

**Affiliations:** ^1^ The Francis Crick Institute, Flow Cytometry Science and Technology Platform London UK

**Keywords:** Assay, clinical, Covid‐19, Cytometry, Development, ELISA, Flow, pandemic, Pipeline, SARs‐CoV‐2, SRL, testing, Validation

## Abstract

In March 2020, with lockdown due to the coronavirus pandemic underway, the Francis Crick Institute (the Crick) regeared its research laboratories into clinical testing facilities. Two pipelines were established, one for polymerase chain reaction and the other for Serology. This article discusses the Cricks Flow Cytometry Science Technology Platform (Flow STP) role in setting up the Serology pipeline. Pipeline here referring to the overarching processes in place to facilitate the receipt of human sera through to a SARs‐CoV‐2 enzyme‐linked immunosorbent assay result. We examine the challenges that had to be overcome by a research laboratory to incorporate clinical diagnostics and the processes by which this was achieved. It describes the governance required to run the service, the design of the standard operating procedures (SOPs) and pipeline, the setting up of the assay, the validation required to show the robustness of the pipeline and reporting the results of the assay. Finally, as the lockdown started to ease in June 2020, it examines how this new service affects the daily running of the Flow STP. © 2020 The Authors. *Cytometry Part A* published by Wiley Periodicals LLC on behalf of International Society for Advancement of Cytometry.

At the beginning of February 2020, the COVID‐19 outbreak in the UK gathered pace and it seemed highly probable that the United Kingdom would follow similar lockdown restriction policies seen in other European countries. The Crick’s Flow Cytometry Science Technology Platform (Flow STP) helped prepare scientists to finish current experiments and store experimental material during lockdown to enable an efficient restart upon the eventual lifting of the restrictions. During this time safety measures were introduced into the Flow STP, including social distancing, that directly reduced the number of instruments available for use. When lockdown was announced in the United Kingdom on the 23rd March 2020 the total number of staff allowed into the Crick was reduced to a core group of key workers. The Flow STP was given key worker status and operated to provide flow cytometry to Crick scientists whose sole focus was now COVID‐19.

In the lead‐up to lockdown communications were sent to scientists to highlight the possible implications of the pandemic on Flow STP operation. There were significant changes in the way the Flow STP operated due to the challenges faced outlined in Table 1.

**Table 1:** Table to highlight the approaches taken to overcome each challenge faced throughout the pandemic and pipeline creation

Challenge	Approach
As a consequence of social distancing staffing levels had to be reduced.	Staff predominantly worked from home unless required to attend the Crick to aid in assay development.
	Other members of the team were furloughed under the government Coronavirus Job Retention Scheme.
	Employees received mandatory weekly COVID‐19 swab tests to confirm suitability to work.
	A designated place was created in the lab where scientists could safely drop off and collect samples to reduce face‐to‐face contact. Users communicated with scientists via online video calling. Use of some instruments in close proximity was restricted, there was reduced occupancy in laboratories and extended cleaning regimes were put in place.
	Training was suspended to prevent any potential spread of infection, and remote support was provided as required.
The strict and constantly changing timelines for the development of both the assays and pipeline.	The Crick worked closely with a UKAS accredited medical laboratory to quickly meet the governance requirements allowing swift assay development.
Introducing the different approach required to work in diagnostics versus research.	The team was able to draw on diagnostic expertise from existing staff members within the Crick with a diagnostic background.
	We consulted with qualified biomedical scientists within the Crick to highlight where processes need adapting to conform to diagnostic standards.
Restrictions imposed by space available to accommodate equipment and staff.	A complete overhaul of both the layout and laboratory equipment was undertaken in less than a week to meet the requirements of a CL2 diagnostic facility.
Balancing the COVID‐19 pipeline with usual workload responsibilities.	Users previously trained in cell sorting were required to perform their own sorts and analysis and were encouraged to help their nontrained colleagues to use the facility with oversight from the STP staff.
	During lockdown non‐essential flow cytometry work was suspended and external users were banned.
Incorporating COVID‐19 compliant practices into training regimes for new users.	Use of PPE including masks and face shields.
	Development of a series of online videos to provide an alternative to face to face contact.

In early May 2020 the Crick prepared to support testing during the pandemic by establishing the Crick COVID‐19 Consortium that successfully developed a diagnostic polymerase chain reaction (PCR) pipeline (1, 2). The Flow STP was engaged to support COVID‐19 research at the Crick, helping to develop novel flow cytometry assays utilizing SARS‐CoV‐2 virus‐specific proteins. The STP was tasked with building a new clinical diagnostic lab and running a novel enzyme‐linked immunosorbent assay (ELISA) that had been developed for detecting antibodies against the S1 spike of the SARS‐CoV‐2 virus. The key processes required are outlined in Figure 1.

**Figure 1:**
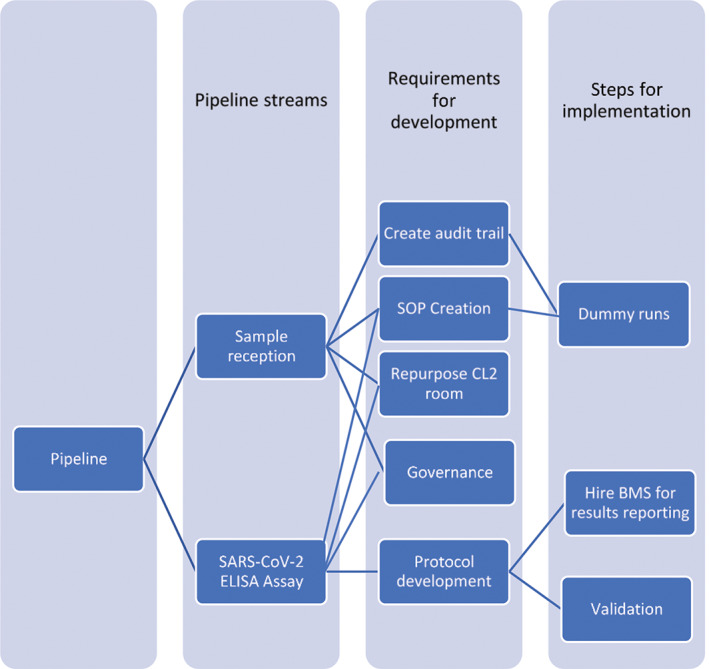
Outline of the key processes involved in pipeline development and implementation.

## SARS‐CoV‐2 assay development

The Flow STP was involved in the development of serology studies, comprising assays, and diagnostic tests focused on antibodies present in serum. Initially, work began on three assays: one cell‐based, one bead‐based, and an ELISA. The early development of these required many steps, beginning with a feasible idea through to procuring and testing reagents and methods. During protocol development testing was required to ensure their precision, accuracy, and coherence.

To accommodate the assay development and pipeline a restricted access containment level 2 (CL2) laboratory was created, housing a ZE5™ (Bio‐Rad, Hercules, CA) and LSR Fortessa™ (BD Biosciences, San Jose, CA), for analysis of serological samples. A second CL2 laboratory was created for ELISA testing.

## the role of governance in diagnostics

To operate as a clinical diagnostic lab specific standards must be attained. The United Kingdom Accreditation Service (UKAS) is a government‐recognized body that provides certified testing, inspection, and calibration services together with an oversight function. UKAS requires medical laboratories to have International Organization for Standardization (ISO) 15189:2012 certification for quality and competence to run a clinical laboratory (3, 4). To meet these standards the Flow STP created new clinical‐grade protocols and standard operating procedures (SOPs). The key requirements are auditability and the ability to connect the samples to their results; these shaped the protocols and the design of the Crick’s new pipeline. The pipeline is in the process of attaining this accreditation.

Other standards and recommendations include those from the Royal College of Pathologists’ “The retention and storage of pathological records and specimens” (5). These outline how to store samples and records required in relation to each sample. In the United Kingdom (and Europe) all personal data must be handled in accordance with data protection laws and regulations (the GDPR and UK Data Protection Act 2018) (6) and all internal recording and sample handling systems must be fit for purpose.

Completing adequate staff training was imperative and staff who built the necessary workflows and resources of the SARS‐CoV‐2 ELISA assay trained other staff to become competent in all processes of the pipeline. Those members of staff trained others until all were fully trained. A training log was kept for each member of staff as part of the audit process and updated when new assays were introduced. The team received information regarding how sample handling adhered to the Human Tissue Act 2004 in the United Kingdom and training in the following areas:

- Good Clinical Practice (GCP) and the legal framework of a diagnostics lab.
- Data protection laws (the GDPR and UK Data Protection Act 2018) (6).

Auditability was achieved through recording processes within the pipeline. Internal quality control (QC) standards for the assays must be kept as specified in the ISO 15189:2012 standard. The following reports were stored and made accessible:

- Instrumentation QC, maintenance history and validation results.
- Reagents QC including validation batch numbers.
- External Quality Assurance (EQA) using a national or internationally recognized body, such as NEQAS in the United Kingdom (7).
- Maintenance and calibration records for equipment for example, pipettes used in the pipeline, must be kept for up to the lifetime of the equipment plus 4 years (5).
- The temperature of fridges and freezers via a sample management system to give an independent secondary recording.

Access to sample material was restricted in the interest of safety, information security, and confidentiality. Other governance comes from the Crick’s institutional policy documents.

## rules on results reporting

The final step of the pipeline was reporting serology test results to a UKAS accredited medical laboratory by a certified Biomedical Scientist (BMS). The BMS’s responsibility is to oversee the process and to ensure the results meet the specific standards set out above. They are given the processed results of the tests, as well as the raw data, to assess the quality and ensure they meet the standards for a clinical virologist to make a diagnosis.

## building a diagnostic pipeline

**Figure 2:**
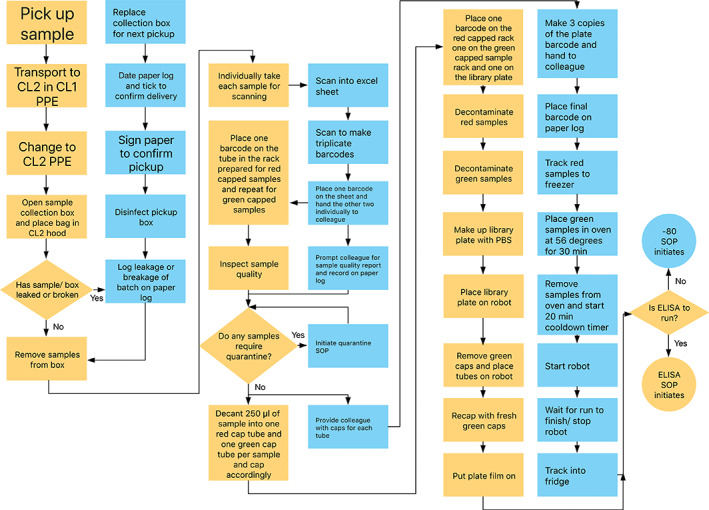
The flowchart outlines the steps taken along the pipeline from the moment of sample receipt to running the assay. The yellow pathway follows the person performing the tasks and making decisions. The blue pathway indicates a second person who checks the action of the person following the yellow pathway, as well as, tracking the sample manually along the pipeline. Rectangles denote actions, diamonds indicate decision points and circles provide the two end points.

The audit trail formed the basis for the development of the sample reception process. At every decision point in designing the pipeline the primary concern was to ensure the sample could not be separated from its result. A two‐person approach, as outlined in Figure 2, was used to enhance the level of security within the decision making and verification processes. SOPs were created to give clear guidelines for how the process should be carried out. These documents will be extended and added to over time.

Logs were created to identify the sample status across the entire pipeline and allowed identification of potential breakpoints along the way. For each breakpoint a contingency plan was developed in case of errors as shown in Table 2. The contingencies were then graded on a traffic light system: red, amber, and green to indicate the level of impact on the outcome of the assay and dictate the level of verification required.

**Table 2:** Demonstrates examples of contingencies in place and their grading in case of operator error or equipment failures throughout pipeline.

Issue	Contingency	Grade
Aspirator breaks.	Use multi‐channel pipette to remove wash.	Green
No precoated plates prepared.	Masterplate stored in fridge at 4°C for up to 72 h.	Green
Barcode scanner breaks.	Manually record the barcode number in the paper log and on the 2 ml tube, proceed as normal.	Amber
Clumpy/hemolyzed sample upon aliquoting.	Record this information in log and continue.	Amber
Plate reader failure.	Add 50 μl of 1 M NaOH to quench plate at 15 min after addition of substrate, store in dark by wrapping in foil at room temperature until problem can be resolved.	Red
	If >12 h repeat assay.	
Any steps not completed as per SOP/missed.	Terminate assay and repeat next day.	Red

As part of this process quarantine measures were introduced ensuring that unscheduled samples that arrived without manifests, or samples with specific rejection criteria were stored in a separate area. This was a location within the fridge that was separated by quarantine criteria notifying the team that the reporting body should be contacted for further instruction before samples were processed. This removed the chance of processing a sample that was not meant to be tested or one that met rejection criteria which would potentially invalidate the result.

As the pipeline developed, samples were received from multiple sources for various uses which required either diagnostic, research based or quality control output channels. The sample reception process was evolved to accommodate this.

## why not automate from the outset?

The pipeline was started manually to get it working as quickly as possible and to enable a more efficient and timely digital development process. The parameters for a Laboratory Information Management System (LIMS) evolved from building the pipeline physically from scratch. Pressure tests in the form of dummy runs with batches of mock samples were undertaken to test the robustness of the manual pipeline. These steps were then used as a framework for digitization as shown in Table 3.

**Table 3:** Breakdown of the steps involved in the pipeline, how each step is manually recorded and the proposed electronic alternative

Task	Manual step	Electronic alternative
Samples received by the Crick from courier.	Sample delivery recorded via Crick usual processes	Notification system alerts
		Flow team that samples have been received
Samples collected from Crick drop off point and transported in the transfer box via shortest route.	Operator signs the paper log to confirm collection of samples	Notification system alerts
		Flow team the samples are in transit to the lab
Sample container placed in hood and visually inspected for leakages.	Operator records if any sample leakages seen on paper log	Operator records if any sample leakages are seen on LIMS
Samples scanned to confirm receipt.	Record made on the paper log of number on box	Notification system alerts sender samples have been received
Quarantine process initiated for any samples that are unexpected, incorrect or missing.	Quarantine logbook on sample fridge requires signing into the fridge with specific location and reason for quarantine.	Fridge Log tracks what samples are in which section of quarantine and notification system alerts sender samples are waiting.
For each sample 250 μl of serum will be transferred into one 5 ml tube and has a bar code attached.	One barcode is stuck to the paper log to confirm which tubes have been received.	Notification system records which samples match those we are expected to receive and highlights any “Quarantine” samples.
Serum quality logged for any hemolyzed or viscous samples.	Any samples that are hemolyzed or viscous are marked next to their barcode on the paper log.	Option on the dashboard to select sample is hemolyzed or viscous for specific samples.
Up to 40 samples are placed onto the robot and duplicated in a 96‐well plate (Library Plate) with one Plate Manifest barcode.	Table filled in with sample barcodes.	The Plate barcode is scanned.
	A copy of the Plate Manifest barcode is attached.	The robot individually scans the barcode of each sample and tracks it into whichever well the sample is placed.
		A plate manifest layout document is created with the date and time.
40 original 5 ml tube samples tracked for −80°C storage and the date and time marked on the rack.	Date and time of the 5 ml samples recorded.	FreezerPro® records date and time the 5 ml samples are being tracked.
Sample tubes are stored in the fridge (4°C) until ELISA is complete.	Operator signs samples into fridge using paper log.	N/A
ELISA process carried out.	SOP checklist double signed to confirm operator has carried out each stage.	Electronic checklist updated when each section of ELISA SOP completed.
ELISA plate placed on reader.	Plate barcode is recorded on paper log.	Scan plate onto reader so that notification system logs plate barcode as “being read”.
Results generated for reporting.	Print a copy of the CSV. File generated by the plate reader and store.	Electronic reporting process carried out via LIMS.
Completed samples are transferred from fridge to freezer.	Sign in and sign out sheet on each fridge detailing what samples are where.	FreezerPro®.

Once all the parameters had been defined and tested automating the process removed multiple sources of potential human error. This increased not only the efficiency of the pipeline by reducing time spent performing manual steps but also freed up time spent by staff performing the tasks.

Initially, we used a commercial electronic sample tracking system already in place at the Crick. After collaborating with the Scientific Computing STP a web app‐based LIMS was developed that encompassed all the specific requirements and parameters the commercial option could not. This custom‐built system relied upon barcoding and robot technology to electronically track the samples through the pipeline. After further rounds of dummy runs and the creation of new SOPs, to include the new LIMS, the pipeline was fully automated leaving the manual system in place as a contingency for any potential point of failure.

## validating the pipeline

Despite the abundance of recent studies on SARS‐CoV‐2 rigorous testing and knowledge of the immune response to the disease was still lacking. This posed a problem with reporting an accurate serological evaluation. Other ways of testing for SARS‐CoV‐2‐specific antibodies were developed including both ELISA and flow cytometry assays with cells and beads. The development of such tests for diagnostic purposes required each variable to be tested and validated. There is currently no gold standard for SARS‐CoV‐2 serological tests so we used different commercial assays to cross validate our internal assays. Although mostly concordant, the different tests showed disparity between some positive samples. The disparity may be due to biological differences or varying assay sensitivities. Importantly, the negatives remained concordant.

Unlike in research, a diagnostic pipeline requires strictly defined protocols to minimize variability and ensure reliable reporting to external parties. The team identified steps in the pipeline where human error could possibly be introduced and incorporated extra checks or contingencies to prevent this. The first obvious step for introduction of human error was in the creation of the 96‐well master plate. As well as human pipetting errors there was potential to introduce mismatches between sample position and sample ID. We therefore automated this step using a robot to decant samples from their tubes into a recorded position on the master plate. As the robot had not been previously used in a diagnostic pipeline several tests and validations had to be done. At least five runs of an ELISA assay with large sample numbers (*n* > 30) were carried out comparing the reproducibility of the robot to human pipetting.

**Figure 3:**
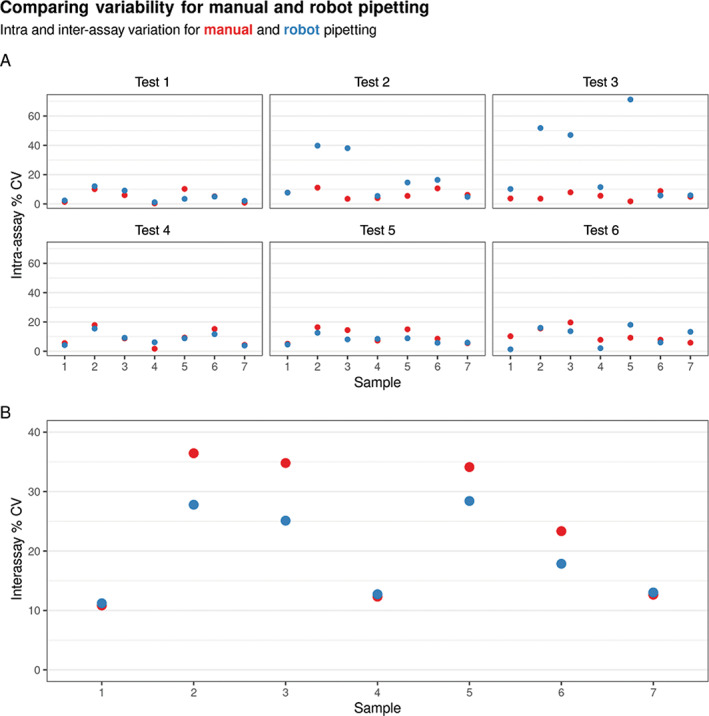
The variability between robot and manual pipetting. (**A**) Intra‐assay percent coefficients of variation (% CV) for each sample across separate subplots for each experimental run. (**B**) Interassay % CV for each sample, collapsed across experimental runs.

**Figure 4:**
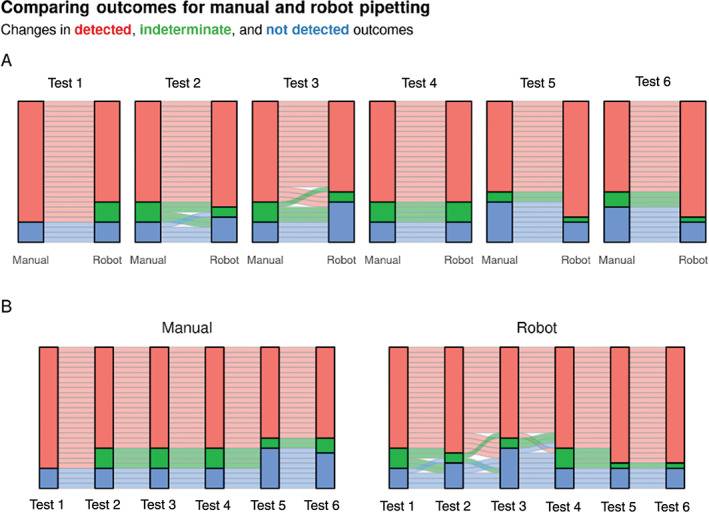
The reproducibility between robot and manual pipetting. (**A**) Alluvial plots showing how the outcome for each well changes when dispensed manually or with the robot. Separate subplots are drawn for each experimental run. Each horizontal ribbon represents a single well and is colored by its manual pipetting outcome. (**B**) The same data as in A showing how the outcome for each well changes between experimental runs. Separate subplots show manual and robot pipetting data.

We calculated the intra and inter percentage coefficients of variation (% CV) to measure the variability of samples both within and between each run. We found that dispensing samples using the robot gave an intra‐assay % CV of 13.07 ± 14.86 (mean ± SD) while human pipetting resulted in an intra‐assay % CV of 7.85 ± 4.74%. Conversely, robotic and human pipetting resulted in interassay % CVs of 19.45 ± 7.51 and 23.50 ± 11.63 respectively as shown in Figure 3. This suggested that while robotic sample dispensing increased intra‐assay variation, believed to be due to uneven volume dispensing, it slightly decreased interassay variation. Importantly, we found the interassay variation in outcomes for each sample (as detected, not detected, or indeterminate) to be lower for robot versus human pipetting as shown in Figure 4. Taken together and with time constraints these data indicated that the assay uncertainty was within reasonable limits and the use of the robot for pipetting resulted in more consistent sample outcomes.

For validation it was necessary to demonstrate specificity, sensitivity and reproducibility within the results of the pipeline (8, 9). Using the ELISA assay as an example we evaluated reproducibility by preparing multiple master plates with different operators. Our test batch of samples contained positives (*n* = 45) and negatives (*n* = 40) as previously confirmed by an independent laboratory through PCR and a commercially available assay. The criterion set for specificity was that tests should not report a negative sample as positive. The criterion for sensitivity was that tests should not report a positive sample as negative. Finally, for reproducibility the criterion was that results should be consistent among the five different repeats. Our results showed the tests were reproducible, specific and sensitive as defined in Table 4.

**Table 4:** Summary of parameters used to validate the performance and suitability of the ELISA assay

	Parameter	Result
1	Reproducibility.	1.18 ± 0.83% of samples had discordant outcomes^a^
2	Sensitivity.	87 ± 3.0%^a^
3	Specificity.	100%^a^
4	Uncertainty.	Intra and interassay % CVs of 8.08 ± 7.23 and 14.57 ± 6.99, respectively^a^
5	Limits of detection.	20.41 pg/ml equivalent of positive control antibody with 95% confidence interval [17.67, 24.65]^b^
6	Dilution linearity.	Linear relationship between log positive control antibody concentration and log absorbance^b^

^a^ Mean ± standard deviation of five runs with 40 samples per run.

^b^ From six independent standard curves of positive control antibody from 0.8 to 200 ng/ml.

**Figure 5:**
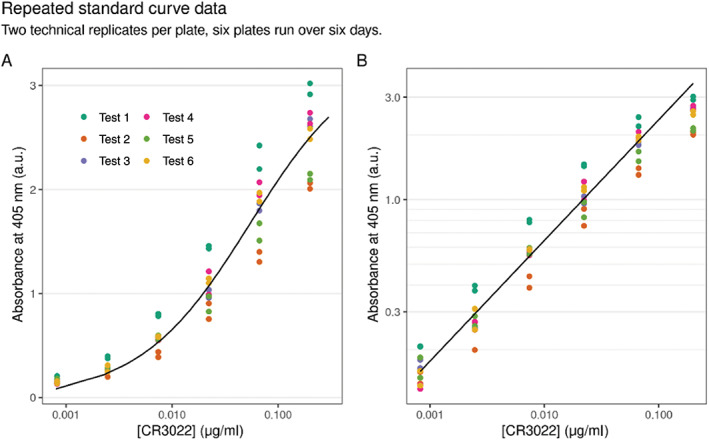
Concentration of the anti‐S1 spike protein antibody CR3022 (the assay positive control antibody) against absorbance at 405 nm after performing six independent ELISAs. (**A**) is plotted on a natural scale and (**B**) is plotted on a semi‐log scale. Two technical replicates per plate are shown. Note that except for the saturating concentration the relationship between log concentration and log absorbance is linear which indicates dilution linearity.

Most diagnostic assays do not include standard curves because they are designed to provide a “YES” or “NO” answer. During the process of pipeline validation however, we included standard curves in some tests. These standard curves provided us with parameters such as limits of detection (20.41 pg/ml equivalent of positive control antibody with 95% confidence interval [17.67, 24.65]) and dilution linearity as shown in Figure 5 and defined in Table 4. Dilution linearity demonstrated that the coating did not interfere with accurate detection or result in nonspecific binding.

While our methods covered all the parameters required for confidence it remained necessary to evaluate each sample individually. It was imperative to evaluate sample stability because assays could be conducted at different time points. For this the same set of samples kept at 4°C were tested at different time points over 2 weeks.

## the new normal

In June 2020 lockdown restrictions were eased; members of the Crick started to return to work in a phased approach and normal staff duties began to increase. The decision was made to split the Flow STP into two groups on a rota basis to enable social distancing and to prevent the entire team having to self‐isolate if one member tested positive for COVID‐19. Careful management of staff time was required taking into consideration both annual and sick leave requirements.

This reduction in staff numbers combined with the running of the clinical diagnostic pipeline had a major impact on the services the Flow STP could provide. The pre‐COVID workload could not be sustained and was streamlined. Prior to the lockdown we had pretrained scientists who were able to perform cell sorting both for themselves and their colleagues. Post lockdown this relieved a large burden on the Flow STP and enabled scientists to continue their research independently. The online booking system was adapted and we implemented a more consultative approach to staff planning and cell sorting which helped triage service requests.

The Flow STP continues to aid researchers with COVID‐19 experiments. As previously discussed flow‐based serological assays continue to be under development internally. These will be added to the clinical diagnostic pipeline based on their success. The reality of the “new normal” for the Flow STP at the Crick is to continue and build on our work on serology assays as a clinical diagnostics lab as well as maintaining an effective flow cytometry service to the Crick research labs. Although our time has been repurposed, putting constraints on our usual duties, we are delighted to contribute our newly adapted services to assist in this global pandemic.

## conclusion

The challenges faced by the Flow STP can be separated into those created by COVID‐19 regarding the changes required to everyday working practices and those posed by the uncertainty and novelty of creating the pipeline as outlined in Figure 1. The practices that have been put in place to overcome the first continue to be instilled in the current climate and are expected to ease off in line with both government advice and updates to Crick policies. While this project would have been possible without it, the team recognizes the advantage of having diagnostic expertise to advise and guide the pipeline development process. Particularly to maximize efficiency by avoiding potential pitfalls. Should the reader be looking to perform a similar process the authors recommend consulting with diagnostic expertise to facilitate this. Incorporating the pipeline into our daily tasks while at reduced staffing levels remains an ongoing challenge. The Flow STP have successfully carried out the development and implementation of a novel SARs‐CoV‐2 ELISA pipeline with the confidence that it is fit for purpose and ready for handover to be maintained outside the Flow STP when required.

## AUTHOR CONTRIBUTIONS

**Emma Russell:** Conceptualization; formal analysis; project administration; validation; writing‐original draft; writing‐review and editing. **Ana Agua‐Doce:** Conceptualization; formal analysis; project administration; validation; writing‐original draft; writing‐review and editing. **Lotte Carr:** Conceptualization; formal analysis; project administration; validation; writing‐original draft; writing‐review and editing. **Asha Malla:** Writing‐original draft; writing‐review and editing. **Kerol Bartolovic:** Conceptualization; writing‐original draft; writing‐review and editing. **Dina Levi:** Writing‐original draft; writing‐review and editing. **Carl Henderson:** Writing‐original draft; writing‐review and editing. **Debipriya Das:** Writing‐original draft; writing‐review and editing. **Hefin Rhys:** Conceptualization; formal analysis; project administration; validation; writing‐original draft; writing‐review and editing. **Philip Hobson:** Conceptualization; formal analysis; project administration; validation; writing‐original draft; writing‐review and editing. **Sukhveer Purewal:** Conceptualization; project administration; writing‐original draft; writing‐review and editing. **Andrew Riddell:** Conceptualization; formal analysis; project administration; resources; writing‐original draft; writing‐review and editing.

## declaration

The following rationale was applied to conclude that no ethics approval was required to conduct this assay development:

- Sera is not HTA relevant material.
- Samples used were leftover serum samples from routine clinical testing of individuals, not taken for a purpose within the remit of Research Ethics Committees.
- Individual samples were anonymized such that no personal identifying information was present.

These samples were provided with appropriate consents from the source.

## Supporting information


**Appendix** **1**. Documentation required for pipeline development including relevant training
