# Supplementary material for: Adapting to the Coronavirus Pandemic: Building and Incorporating a Diagnostic Pipeline in a Shared Resource Laboratory
Source: Cytometry A. 2020 Nov 22;99(1):90–9. doi: 10.1002/cyto.a.24248 (PMC7894326; doi:10.1002/cyto.a.24248)
Supplement: Supplementary file 1 — Appendix 1. Documentation required for pipeline development including relevant training [file CYTO-99-90-s001.doc]

**Appendices**

Appendix 1. Documentation required for pipeline development including relevant training

| Sample reception SOP |
| --- |
| SARS-CoV-2 ELISA SOP |
| Transfer to serum to master plate via robot SOP |
| Manual log for sample tracking |
| Contingency plan document |
| Internal Human Tissue Act 2004 training seminar |
| Internal GDPR training videos and quiz |
| Staff training log document |
